# Tacrolimus Ointment in Periorbital Atopic Dermatitis

**DOI:** 10.7759/cureus.53055

**Published:** 2024-01-27

**Authors:** Mazen Alzahrani, Yumna F Kamal, Muhammad A Akram

**Affiliations:** 1 Ophthalmology, Jeddah Eye Hospital, Jeddah, SAU; 2 Medicine and Surgery, King Abdulaziz University, Jeddah, SAU; 3 Ophthalmology, Sligo University Hospital, Sligo, IRL

**Keywords:** atopic dermatitis, vernal keratoconjunctivitis (vkc), topical tacrolimus, immunomodulatory, topical corticosteroid

## Abstract

Periorbital atopic dermatitis (AD) is a common sign in ophthalmological practice and usually has a persistent and relapsing course. Treatment with topical corticosteroids has various side effects associated with their usage. Tacrolimus topical ointment has unique immunomodulatory properties that decrease skin inflammation and pruritus in AD. In this case series, we present a prospective case series of five patients (three males and two females) who received topical application of tacrolimus ointment 0.1-0.03% in the periorbital area twice daily for one to four weeks. The pre- and post-treatment images of all patients were recorded to compare the effects of the treatment. The cases were selected from patients attending the outpatient clinics of East Jeddah Hospital, Saudi Arabia. All patients were suffering from AD. Patients underwent a clinical assessment by tactile inspection (location, size, color, and surface condition) in the first week, secondweek, third month, and first year. We may conclude from this study that tacrolimus showed promising outcomes and is safe and effective for the treatment of flares or resistant periorbital AD in both adults and children.

## Introduction

Periorbital atopic dermatitis (AD) is a common problem in the fields of dermatology and ophthalmology. AD is a common sign in ophthalmological practice and is usually challenging to treat. It is a type of dermatitis that can be clinically predicted by the presence of red and scaly papules, pustules, and sand swelling in the surrounding area of the eye [1]. The prevalence of atopic dermatitis in children and adults in developed countries is estimated to be 10% and 20%, respectively [2]. Many other disorders occur in the periorbital region with a significantly similar clinical appearance to periocular eczema; hence, it may be misdiagnosed, especially among ophthalmologists [3].

Atopic keratoconjunctivitis might also be seen in patients with AD because eyelid dermatitis causes variations in the sensitivity, anatomy, and functioning of the eyelid [4]. Atopic keratoconjunctivitis results in conjunctival hyperemia with possible corneal disorders such as scarring, persistent epithelial defects, superficial punctate keratitis neovascularization, and papillary hypertrophy [5]. AD has a continuous and relapsing course; thus, atopic periorbital or eyelid disease might render the patient visually disabled.

Corticosteroid ointment is used to treat this condition, but there are various limitations to the prolonged use of cortisone. For instance, prolonged exposure of the sensitive eye surface to steroids can result in the appearance of telangiectatic vessels and skin atrophy [6]. Furthermore, patients exposed to topical corticosteroids were not able to achieve long-term results and were prone to various other adverse effects associated with steroids (glaucoma, rebound phenomenon, cataract) [7,8].

Tacrolimus, a calcineurin inhibitor, has unique immunomodulatory properties that can decrease swelling of the skin and pruritus in AD by blocking T-cell activation and binding to the cytosolic immunophilin receptor, mast cells, and keratinocytes [9]. It is a topical immunomodulatory agent that has been approved by the United States Food and Drug Administration to cure AD in patients with mild-to-severe symptoms. Various studies suggest and emphasize the impact of tacrolimus in treating AD [6,10-12]. In addition, tacrolimus is effective for atopic keratoconjunctivitis patients who are refractory to conventional medications, including topical cyclosporine [13,14].

In this case series, we discuss our practice of applying tacrolimus ointment for the treatment of periorbital AD manifestations and its impact on disease control.

## Case presentation

Case one

**Figure 1:**
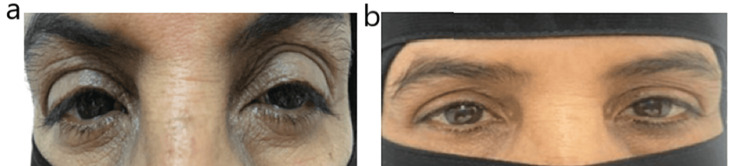
A 56-year-old with a history of diabetes mellitus and periorbital atopic dermatitis. (a) Before Application of ointment. (b) After application of ointment.

A 56-year-old female with a known history of diabetes mellitus complained of inflammation, redness, and scaly skin of the eyelid for four years. She had been using topical corticosteroids often throughout this time, but the condition did not resolve. A thorough examination and assessment of the eyes showed scaly and thickened skin in the periorbital region (Figure 1a). She had been maintaining adequate hygiene, along with oral intake of doxycycline and the application of corticosteroid ointment; however, the swelling worsened over time. We prescribed topical tacrolimus (0.1%) ointment on the periorbital region for around one to four weeks. The ointment was applied twice daily after cleaning the eyelid. Within two weeks of application, improvement in the symptoms was observed. Eventually, she was able to reduce the frequency of treatment. After one year, she was examined and found asymptomatic for AD (Figure 1b).

Case two

**Figure 2:**
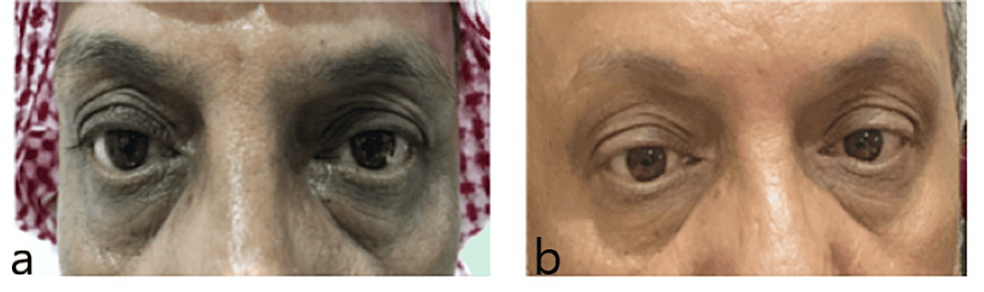
A 60-year-old with a history of hypertension and periorbital atopic dermatitis. (a) Before application of ointment. (b) After application of ointment.

A 60-year-old male suffering from asthma and non-ophthalmic atopic skin disease reported pain and redness in the periorbital region for four years (Figure 2a). Moreover, the periorbital skin and eyelids were scaly in both eyes. His previous medication was changed, and he was instructed to apply tacrolimus (0.1%) ointment on the periorbital skin twice a day. Surprisingly, within just one week of treatment initiation, inflammation was significantly reduced. After using tacrolimus for approximately two weeks, he became asymptomatic (Figure 2b).

Case three

**Figure 3:**
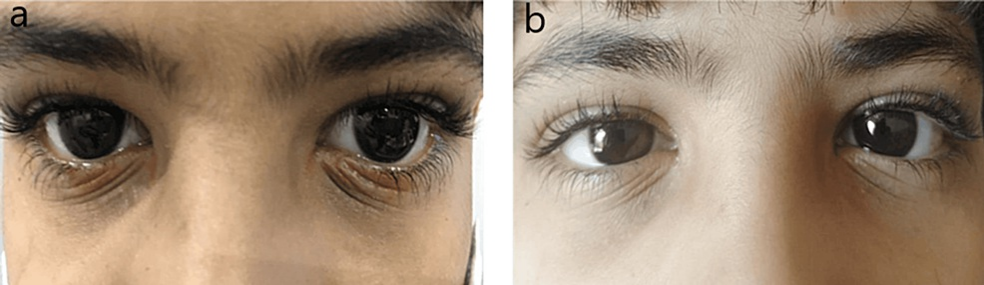
A 10-year-old with a history of periorbital atopic dermatitis. (a) Before application of ointment. (b) After application of ointment.

A 10-year-old patient suffering from periorbital AD showed visible scaly skin of the periorbital region and some thickening of the skin (Figure 3a). The patient’s symptoms were very severe. We suggested the application of tacrolimus ointment (0.03%) for two weeks twice a day. The improvement in the patient was prominent in just a few days. On assessment after one month, his symptoms had completely resolved (Figure 3b).

Case four

**Figure 4:**
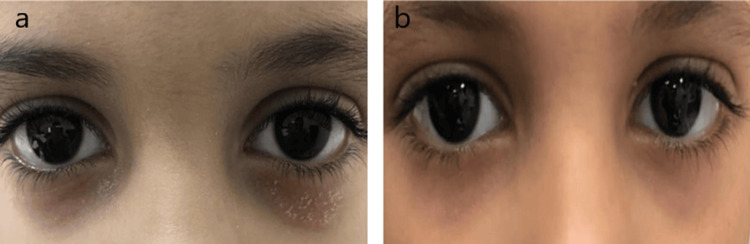
An eight-year-old with a history of periorbital atopic dermatitis. (a) Before tacrolimus ointment application. (b) After tacrolimus ointment treatment.

An eight-year-old boy was suffering from periorbital AD. Along with inflammation, there was redness and disruption of the skin under the eyes (Figure 4a). The patient was subjected to various medications for one month, without any clinical improvement. After a clear examination of the case, tacrolimus (0.03%) was advised for two weeks. Significant improvement was observed just after the first week of usage. Previously, the eyelid was thick and bulging, with crusts seen on the lower periorbital region. After one week of application, there was a clear reduction in the thickness and redness of the skin surface (Figure 4b). Moreover, the crusts around the skin also disappeared. We continued the ointment application for another week, following which the patient became asymptomatic.

Case five

**Figure 5:**
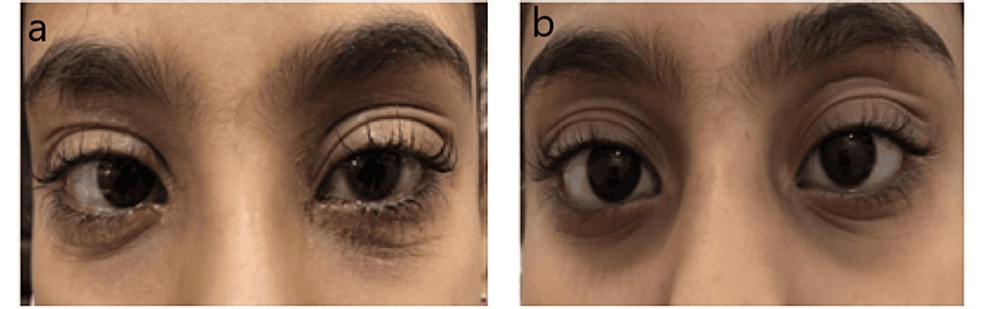
A 12-year-old with a history of vernal keratoconjunctivitis and periorbital atopic dermatitis. (a) Before application of ointment. (b) After application of ointment (right).

A 12-year-old girl had combined symptoms of vernal keratoconjunctivitis (VKC) and AD. In addition to inflammation and redness, there was exudation from both eyes with continuous itching, irritation, and pain (Figure 5a). She was given tacrolimus ointment (0.03%) for two weeks. There was an improvement in her condition following treatment (Figure 5b).

## Discussion

In the current case series, we selected five patients from the outpatient department of East Jeddah Hospital. These patients included both males and females belonging to different age groups, ranging from eight to 60 years. These patients had symptoms of red and scaly eyelids, itchiness, and a history of periorbital dermatitis. Upon thorough assessment, some patients had papillary reactions in conjunction with ocular AD. All patients were previously using hydrocortisone without any permanent or proper improvement.

We prescribed topical tacrolimus 0.03-0.1% ointment twice a day for one to four weeks to all patients, following current practice [15]. The patients were examined during the first week, second week, third month, and first year. In concordance with previous reports, all patients in our study showed dramatic improvements in inflammatory signs and symptoms without significant adverse effects. All patients completed 12 months of follow-up, and none required additional medications, such as antihistamines, steroids, or mast cell stabilizers, to control disease activity.

In a multicenter, randomized clinical trial, Ohashi et al. [14] used tacrolimus 0.1% ophthalmic suspension twice daily for four weeks in five patients with VKC and compared the outcome to a placebo group. They found the treated eyes showed a marked improvement in symptoms after four weeks of treatment.

The most frequent ointment side effect is a burning sensation at the application site; hence, it is essential to advise patients to use sunscreen and minimize exposure to sunlight to avoid its carcinogenic effect [16]. In our study, patients did not complain of any adverse effects of tacrolimus; however, this may be due to the small sample.

The symptoms of most patients in our study were relieved one to two weeks after beginning tacrolimus ointment treatment. In addition, allergic symptoms did not recur once treatment was started, and as the frequency of dosage was reduced, a longer follow-up period is required to accurately assess recurrence.

Although a risk of T-cell lymphoma in patients using topical tacrolimus has been reported [17], there is insufficient epidemiological evidence to determine if topical calcineurin inhibitors can cause malignancy [18]. Moreover, data are scarce regarding the optimal dose and duration of treatment.

In our study, no malignancies occurred during the one-year follow-up period, and the risk of developing malignancy after the application of topical tacrolimus 0.1% ointment was extremely low.

Topical tacrolimus proved to be effective in the short-term cure of AD and VKC through a random placebo-controlled approach. The results of tacrolimus were also verified by a clinical trial in which 632 patients were observed for the effects of tacrolimus ointment (0.03-0.1%) [12].

The patients given tacrolimus ointment in our study showed better improvement in symptoms of skin eczema compared to when they were treated with corticosteroids.

Moreover, the application of this ointment does not interact with the metabolism of collagen at the targeted site, thereby preventing skin atrophy [10]. In addition, tacrolimus reduces the risk of discoloration of the skin and telangiectatic vessels, which is a possible side effect of topical corticosteroids [5].

A significant improvement in the symptoms of topical dermatitis was seen, which could be suggestive of further clinical trials and the safe recommendation of tacrolimus in clinical practice. Patients should be assessed for the recurrence of disease symptoms at least once a year. The best results of the usage of tacrolimus ointment are obtained with regular use, without any negligence.

Topical tacrolimus is to be used on the skin surface, but patients experienced a significant improvement in diseases associated with ophthalmic surfaces. However, the best application strategy, whether to be applied in the tear film or through regional adsorption, is still to be evaluated for the best functioning and anatomy of the periocular region.

## Conclusions

Studies have revealed the effectiveness of topical tacrolimus ointment for the treatment of flares or resistant periorbital dermatitis in adults and children. In this case series, we tried to emphasize the effect of tacrolimus ointment in curing atopic keratoconjunctivitis and AD. Excellent results were obtained with the application of topical tacrolimus ointment within a short duration of one to two weeks. Therefore, the application of topical tacrolimus has proved to be suitable for AD and has an additional advantage for patients with atopic keratoconjunctivitis. After a one-year follow-up, no side effects were seen. However, prolonged usage of tacrolimus ointment may lead to side effects that must be carefully analyzed during usage.
